# The Ratio of Oxygen Uptake From Ventilatory Anaerobic Threshold to Respiratory Compensation Point Is Maintained During Incremental Exercise in Older Adults

**DOI:** 10.3389/fphys.2022.769387

**Published:** 2022-03-03

**Authors:** Kazuyuki Kominami, Keiko Imahashi, Toko Katsuragawa, Mitsuyo Murakami, Masatoshi Akino

**Affiliations:** ^1^Cardiac Rehabilitation Center, Sapporo Ryokuai Hospital, Sapporo, Japan; ^2^Department of Cardiovascular Medicine, Sapporo Ryokuai Hospital, Sapporo, Japan

**Keywords:** isocapnic buffering phase, ventilatory anaerobic threshold, respiratory compensation point, aging, oxygen uptake, cardiopulmonary exercise testing

## Abstract

**Introduction:**

The period from ventilatory anaerobic threshold (VAT) to respiratory compensation point (RCP) during incremental exercise (isocapnic buffering phase) has been associated with exercise tolerance and skeletal muscle composition. However, several reports compare younger and older healthy adults, and specific age-related changes are unclear. This study aimed to examine the oxygen uptake (VO_2_) from VAT to RCP and its change over time in younger and older healthy adults.

**Methods:**

A total of 126 consecutive participants were divided into two groups (95 younger and 31 older than 50 years of age) who underwent cardiopulmonary exercise testing, and VAT and RCP were determined. The ratio (RCP/VAT) and difference (ΔVO_2_ RCP-VAT) were calculated from the VO_2_ of VAT and RCP and compared between groups and ages. Statistical analyses included *t*-tests and Spearman’s correlation tests, and the significance level was set at <5%.

**Results:**

RCP/VAT was not significantly different (1.40 ± 0.19 vs. 1.59 ± 0.24, *p* = 0.057) but weakly correlated with age (*r* = −0.229, *p* = 0.013, *y* = −0.0031x + 1.7588, lowering rate: 0.185%/year). Conversely, ΔVO_2_ RCP-VAT was significantly lower in the older group (7.7 ± 3.1 vs. 13.8 ± 4.9 ml/kg/min, *p* < 0.001) and correlated significantly with age (*r* = −0.499; *p* < 0.001; *y* = −0.1303x + 16.855; lowering rate, 0.914%/year).

**Conclusion:**

ΔVO_2_ RCP-VAT was considered to be a poor indicator of lactate buffering capacity in the IB phase because both VAT and RCP were greatly affected by age-related decline. Conversely, RCP/VAT was suggested to be an index not easily affected by aging.

## Introduction

In cardiac rehabilitation settings, exercise tolerance is generally assessed by cardiopulmonary exercise testing (CPET) using a combination of incremental exercise testing and expiratory gas analysis (Mann et al., 2013; Mezzani et al., 2013; JCS Joint Working Group, 2014; Price et al., 2016). The ventilatory anaerobic threshold (VAT) is the point at which carbon dioxide excretion (VCO_2_) increases in response to an increase in oxygen uptake (VO_2_) (Sullivan and Cobb, 1990; Nishijima et al., 2017, 2019). The respiratory compensatory point (RCP) is the point at which the exercise intensity increases and excessive carbon dioxide excretion by respiratory compensation begins due to accumulation of H^+^ ions (Balady et al., 2010). The RCP is partially dependent on the chemosensitivity of the carotid bodies and the accumulation of lactate during incremental exercise (Takano, 2000). Between the VAT and RCP is the isocapnic buffering phase (IB phase) in which the increased lactate is buffered by bicarbonate (Beaver et al., 1986a; Wasserman et al., 2012; Yen et al., 2015).

During the IB phase, oxygen uptake increases relative to ventilation (VE/VO_2_); however, carbon dioxide excretion does not (VE/VCO_2_). The IB phase is a period of lactate buffering activity because the lactate produced by exercise is buffered and utilized *in vivo*; it is therefore thought to be related to the lactate steady state without rapid acidosis (Dekerle et al., 2003; Keir et al., 2018; Iannetta et al., 2019). In athletes, the IB phase is further associated with maximal oxygen uptake, a criterion for exercise tolerance (Korkmaz Eryılmaz et al., 2018), and patients with coronary artery disease show a similar relationship (Yen et al., 2018). However, it has been reported that the IB phase is shorter in older individuals (Lenti et al., 2011).

In previous reports, the time in the IB phase (Tanehata et al., 1999; Nakade et al., 2019) and ΔVO_2_ RCP-VAT (Lenti et al., 2011; Carriere et al., 2019) have been used to define the IB phase; however, exercise tolerance indices such as peak VO_2_ decline with age. The time can only be compared with the same protocol [it may be possible to adjust it; however, an adjustment is not desirable because of the errors in the load acceleration and oxygen uptake (Hansen et al., 1987)]. Thus, ΔVO_2_ can be used to compare similar groups or those within the same age group; however, changes cannot be considered due to aging. It is not clear whether the shortening and decline in the indices of the IB phase used in the past are characteristically caused by aging or to what extent the decline is due to age-related changes. In the present study, we hypothesized that the ratio of oxygen uptake between RCP and VAT could be used as an indicator of IB phase because it can show how much oxygen uptake increases from VAT to RCP. By assessing the ratio of RCP to VAT, it would also be possible to easily show the lactate buffering capacity, and if it is the ratio of RCP to VAT oxygen uptake, it would be possible to make comparisons between ages.

Therefore, this study aimed to use the results of CPET to identify differences and changes over time in the IB phase using RCP/VAT and ΔVO_2_ RCP-VAT in healthy younger and older participants.

## Materials and Methods

There were 126 healthy adult participants who completed a symptom-limited CPET in our hospital, for whom the VAT and RCP could be identified. Participants of previous studies (Kominami et al., 2015, 2021) and those who underwent screening tests at our hospital were enrolled; none of them had any diseases or had been treated with medications for indications such as cardiovascular or respiratory diseases. They were divided into two groups: <50 years (*n* = 95) and >50 years of age (*n* = 31). The participants’ characteristics are presented in Table 1.

**TABLE 1:** Participants’ clinical characteristics.

		All	Young (<50 years)	Older (≥50 years)
		*n* = 126	*n* = 95	*n* = 31
Age	(years)	35.5 ± 20.5	24.0 ± 6.3	68.6 ± 6.5*
Sex	(male:female)	88:38	71:24	17:14
Height	(cm)	167.5 ± 8.9	170.0 ± 8.2	160.0 ± 6.1*
Body weight	(kg)	62.7 ± 10.7	63.8 ± 10.6	59.5 ± 10.5
BMI		22.3 ± 2.8	22.0 ± 2.7	23.1 ± 3.1

*Data are presented as mean ± standard deviation. BMI, body mass index.*

*Significant (p < 0.05) for younger vs. older group.

### Exercise Testing

Cardiopulmonary exercise testing was performed using a stationary bicycle (StrengthErgo 8; Mitsubishi Electric Engineering, Tokyo, Japan) and a breath-by-breath gas analyzer (AE-300S; Minato Ikagaku Co., Tokyo, Japan). Symptomatic maximal exercise was performed using a ramp protocol of 5–30 watts (W)/min according to age and condition after 2–3 min rest and warm-up of 0–10 W lasting 2–3 min. Rating of perceived exertion (RPE) at the end of the exercise was assessed using the Borg scale (Beaver et al., 1986a; Takano, 2000; Dekerle et al., 2003; Balady et al., 2010; Lenti et al., 2011; Wasserman et al., 2012; Yen et al., 2015, 2018; Nishijima et al., 2017, 2019; Keir et al., 2018; Korkmaz Eryılmaz et al., 2018; Carriere et al., 2019; Iannetta et al., 2019; Nakade et al., 2019).

### Expiratory Gas Analysis Index

#### Ventilatory Anaerobic Threshold

The VAT was visually determined using the modified V-slope method as described by Sue et al. (1988), which is a modification of the method described by Beaver et al. (1986b).

The ventilatory equivalent method (the point at which VE/VO_2_ begins to rise without an increase in VE/VCO_2_) and end-tidal methods (PetO_2_ begins to rise without a decrease in PetCO_2_) was used as a complement (Balady et al., 2010; Wasserman et al., 2012).

#### Respiratory Compensation Point

Respiratory compensation point was comprehensively determined from the point where PetCO_2_ decreased, VE/VCO_2_ began to increase, and the inflection point of the VE/VCO_2_ slope.

**FIGURE 1:**
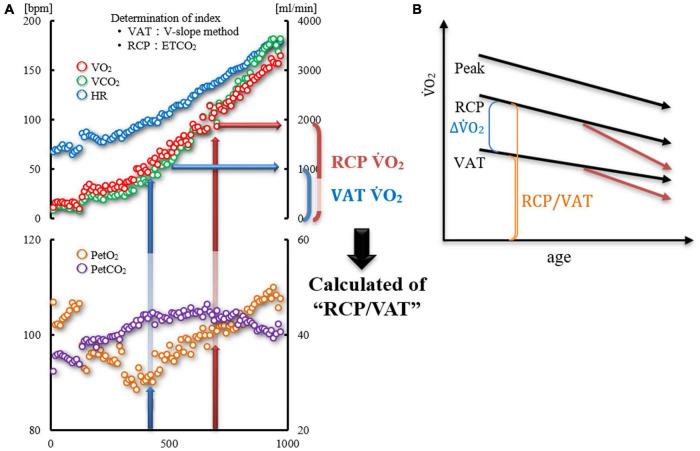
**(A,B)** Diagram showing the calculation of “RCP/VAT”. **(A)** Time trends of HR, VO_2_, VCO_2_, PETO_2_, and PETCO_2_ in one case. VAT was determined using the V-slope method and RCP from PETCO_2_ and VE/VCO_2_ slope. The RCP/VAT was calculated by dividing the RCP VO_2_ by the VAT VO_2_. **(B)** Age-related changes of VAT, RCP, and Peak VO_2_ (three black allows). It is believed that the decline in VAT is slower than that in RCP due to aging. Therefore, ΔVO_2_ RCP-VAT decreases with age; however, RCP/VAT may not change with age. If a disease associated with chronic inflammation, such as heart disease, develops, both VAT and RCP may further decrease, as shown by the red arrows, as well as ΔVO_2_ RCP-VAT and RCP/VAT.

The values of VAT VO_2_ and RCP VO_2_ were used to calculate RCP/VAT and ΔVO_2_ RCP-VAT (Figures 1A,B).

#### VE/VCO_2_ Slope

VE/VCO_2_ slope as ventilation efficiency during incremental exercise load was calculated as the slope of linear regression from the start of exercise to RCP.

#### Oxygen Uptake Kinetics (ΔVO_2_/ΔWork Rate)

The VO_2_–work rate relationship during ramp exercise testing was evaluated by plotting the work rate on the x-axis and VO_2_ on the y-axis (Hansen et al., 1987; Wasserman et al., 2012). Both maximal and submaximal exercise data were plotted on the same graph. The initial time delay was removed from the analysis (Hansen et al., 1987). Each slope was calculated using linear regression for the maximum tests.

### Statistical Analysis

Data are presented as the mean ± standard deviation. Statistical analyses were performed using Statistics for Excel 2012 (Social Survey Research Information Co., Tokyo, Japan). Student’s *t*-test was used for comparisons between the two groups. Pearson’s correlation coefficient was used to determine the correlation between age and each parameter, and the lowering rate with age was calculated from the regression coefficient of the regression line. The 95% confidence intervals and prediction intervals were also calculated for the relationship between age and RCP/VAT, ΔVO_2_/weight RCP-VAT, VAT VO_2_/weight, and RCP VO_2_/weight. The significance level was set at 5%.

### Ethical Considerations

This research was conducted in accordance with the code of ethics of Sapporo Ryokuai Hospital and with due consideration for the protection of the participants’ personal information. Informed consent was obtained from all participants for their participation in the study and for publication of this report. The data obtained were de-linked and anonymized, and this study was conducted using the data for analysis. The authors confirmed that all participants could not be identified and that they were fully anonymized. Furthermore, the authors affirm that all mandatory health and safety procedures were complied within the course of conducting any experimental work reported in this paper.

## Results

No adverse events such as arrhythmia, angina pectoris, or worsening of heart failure requiring treatment occurred during CPET. Regarding the characteristics of the participants, there were no significant differences in sex, weight, or BMI (Table 1).

### Cardiopulmonary Exercise Testing Parameters

Each parameter in the CPET is listed in Table 2. Compared with the younger group, the older group showed significantly lower values of VAT and RCP VO_2_ (*p* < 0.001, respectively). Moreover, PetCO_2_ was significantly lower in the elderly group, and the VE/VCO_2_ slope was significantly higher (*p* < 0.001, respectively).

**TABLE 2:** Primary cardiopulmonary data during exercise.

		All	Young (<50 years)	Older (> = 50 years)	Effect size (r)
		*N* = 126	*N* = 95	*N* = 31	
Peak VO_2_	(ml/min)	2173 ± 714	2434 ± 609	1390 ± 327*	0.626
Peak VO_2_/weight	(ml/kg/min)	34.4 ± 9.0	38.0 ± 6.9	23.6 ± 4.7*	0.692
Peak HR	(bpm)	168 ± 21	178 ± 11	140 ± 20*	0.776
Peak R		1.22 ± 0.10	1.24 ± 0.10	1.17 ± 0.08	0.297
RPE; dyspnea		15.5 ± 2.1	15.9 ± 1.8	14.1 ± 2.4*	
RPE; leg fatigue		17.1 ± 1.8	17.5 ± 1.4	15.7 ± 2.3*	
VAT VO_2_	(ml/min)	1058 ± 336	1161 ± 315	746 ± 153*	0.539
VAT VO_2_/weight	(ml/kg/min)	16.9 ± 4.4	18.3 ± 4.0	12.7 ± 2.6*	0.542
VAT HR	(bpm)	112 ± 18	119 ± 15	93 ± 11*	0.632
VAT R		0.86 ± 0.06	0.86 ± 0.06	0.87 ± 0.05	0.096
RCP VO_2_	(ml/min)	1758 ± 544	1925 ± 486	1170 ± 253*	0.578
RCP VO_2_/weight	(ml/kg/min)	29.3 ± 7.9	32.0 ± 6.3	20.0 ± 5.5*	0.610
RCP HR	(bpm)	149 ± 22	158 ± 15	119 ± 17*	0.730
RCP R		1.06 ± 0.06	1.07 ± 0.06	1.03 ± 0.06	0.218
RCP/VAT		1.65 ± 0.27	1.68 ± 0.28	1.56 ± 0.21	0.205
ΔVO_2_/ΔWR		10.7 ± 1.0	10.7 ± 1.0	10.8 ± 1.0	0.050
VE/VCO_2_ slope		23.8 ± 4.0	22.3 ± 2.9	28.1 ± 3.6*	0.629
PetCO_2_ at rest	(mmHg)	36.4 ± 3.6	37.4 ± 3.4	33.5 ± 2.6*	0.470
PetCO_2_ at VAT	(mmHg)	44.1 ± 4.4	45.6 ± 3.7	39.5 ± 3.2*	0.598
PetCO_2_ at RCP	(mmHg)	45.3 ± 5.2	46.7 ± 4.5	40.1 ± 4.0*	0.533
PetCO_2_ RCP – rest	(mmHg)	8.8 ± 3.6	9.3 ± 3.6	6.6 ± 2.6*	0.317
VD/VT at rest	(%)	39.7 ± 4.9	38.5 ± 4.9	42.9 ± 3.1*	0.395
VD/VT at VAT	(%)	30.2 ± 4.5	28.6 ± 3.5	34.9 ± 3.6*	0.618
VD/VT at RCP	(%)	25.5 ± 3.9	24.1 ± 2.9	30.1 ± 3.3*	0.645
VD/VT RCP – rest	(%)	−13.9 ± 4.3	−14.3 ± 4.4	−12.7 ± 3.5	0.160

*Data are presented as mean ± standard deviation. VO_2_, oxygen uptake; VCO_2_, carbon dioxide; VE, minute ventilation; PETCO_2_, end-tidal carbon dioxide pressure; VD/VT, dead-space gas volume to tidal volume ratio; AT, anaerobic threshold; RCP, respiratory compensation point; HR, heart rate; RPE, rating of perceived exertion; WR, work rate; R, respiratory exchange rate.*

*Significant (p < 0.05) for younger vs. older group.

Furthermore, both ΔVO_2_/weight RCP-VAT and RCP/VAT showed a significant correlation with Peak VO_2_ (*r* = 0.629, *p* < 0.001 and *r* = 0.217, *p* = 0.017, respectively).

### Respiratory Compensation Point/Ventilatory Anaerobic Threshold and ΔVO_2_/Weight Respiratory Compensation Point-Ventilatory Anaerobic Threshold

**FIGURE 2:**
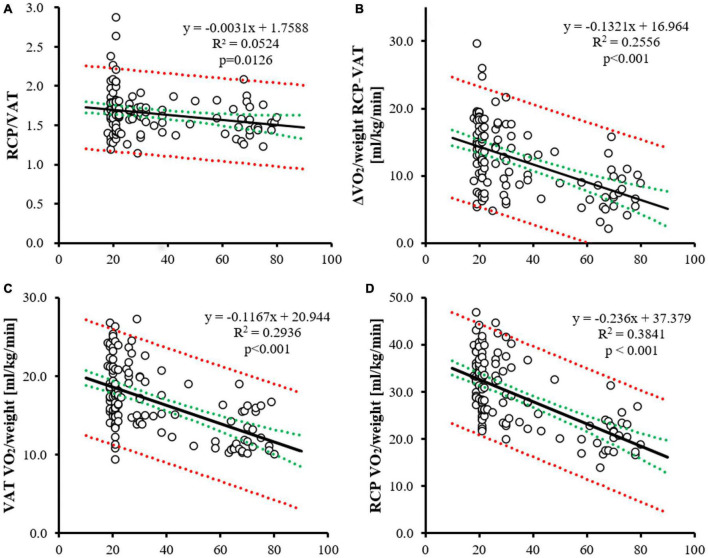
Age–related change of RCP/VAT, VO_2_/weight RCP-VAT, VAT and RCP VO_2_/weight. The age-related change of RCP/VAT **(A)**, VO_2_/weight RCP-VAT **(B)**, VAT **(C)** and RCP VO_2_/weight **(D)**. The horizontal axis indicates age. For each panel, the correlation coefficient (linear regression equation and lowering rate per year; black line), 95% confidence intervals (red dotted line), and prediction intervals (green dotted line) are shown.

There was no significant difference in RCP/VAT between the younger and older groups (Table 2 and Figure 2). The coefficient of the linear regression equation was −0.0031, and the annual rate of decline was 0.185%, indicating that the effect of aging was not significant. ΔVO_2_/weight RCP-VAT was significantly lower in the older group, and age-related changes showed moderate negative correlation. The coefficient of the linear regression equation was −0.1303, and the annual rate of decline was 0.914%, indicating a significant effect of age-related changes.

## Discussion

In this study, IB during incremental exercise load was expressed as a ratio of oxygen uptake, which is different from that in previous studies. Moreover, the differences between healthy younger and elderly subjects and the changes over time were investigated. During the IB period from VAT to RCP, as the exercise intensity increases, lactate is buffered by HCO_3_^–^. Thus, a longer IB phase is less likely to lead to metabolic acidosis, allowing for the high-intensity exercise to continue for longer. Conversely, a short IB phase or an early onset of RCP may be associated with shortness of breath and fatigue. Our results show that the ratio of oxygen uptake during IB (RCP/VAT) is not significantly different in healthy older adults compared to healthy younger adults and is a modest indicator compared to the decline in VAT, RCP, and ΔVO_2_ RCP-VAT.

### Age-Related Changes in Respiratory Compensation Point/Ventilatory Anaerobic Threshold

In this study, the decrease in VAT and RCP VO_2_/weight was −8.8 and −16.2 ml/min/year (−0.118 and −0.242 ml/kg/min/year, 0.627 and 0.722%/year), respectively, which was comparable to that reported in previous studies (Itoh et al., 2013). Similarly, the ΔVO_2_/weight RCP-VAT decreased with age. VAT is detected in CPET by the excretion of carbon dioxide in the exhaled air due to the buffering of lactate, which is produced by increased glycolysis associated with increased exercise intensity. The presence of increased lactate in the blood and carbon dioxide excretion in the exhaled air is well correlated, though there is a time delay. This association is derived from the proportion of slow-twitch fibers in skeletal muscle and CO_2_ storage *in vivo* (Ivy et al., 1980). The RCP is the period from the VAT through the IB phase to the onset of respiratory compensation. The buffering capacity of CO_2_/H^+^ produced by lactate is influenced by the fiber type in skeletal muscle; previous studies have shown an association with skeletal muscle composition, particularly type 2 fibers (Nakagawa and Hattori, 2002).

Several mechanisms are believed to allow the organism to rapidly maintain homeostasis in response to dynamic exercise (Bruce et al., 2019). The excretion of carbon dioxide during expiration is related to pulmonary blood flow, that is, cardiac output and ventilatory capacity. In our study, PETCO_2_, a measure of cardiac output, was significantly lower in the older group (though cardiac output was not measured directly and may therefore be dissociated from actual measurements). Additionally, the VE/VCO_2_ slope was higher in the older group, which may have contributed to the reduced capacity for lactate buffering and carbon dioxide excretion up to RCP, and the higher rate of decline in RCP VO_2_ than VAT with age.

In addition, ΔVO_2_/ΔWR, which indicates the oxygen utilization capacity of the peripheral motor muscle group, was not affected by age. In patients with specific risk factors, the reduced oxygen availability of the peripheral motor musculature facilitates anaerobic energy production during exercise, leading to the production of lactic acid and the buffering and excretion of CO_2_. In patients with heart failure, a decrease in cardiac output and vasodilatory capacity limits blood flow to peripheral exercise muscle groups, leading to a decrease in ΔVO_2_/ΔWR. In the present study, ΔVO_2_/ΔWR was not affected by aging, suggesting that the effect on energy production was small. However, we were not able to investigate related factors such as circulating blood volume, total body skeletal muscle mass, muscle composition, and plasma bicarbonate ion concentration concerning the accumulation and buffering capacity of CO_2_/H^+^ produced *in vivo*.

The results of this study showed that various indices of exercise (e.g., peak VO_2_ and VAT) decreased with age, and the rate of decrease was higher for RCP than for VAT. RCP/VAT, the ratio of RCP VO_2_ to VAT VO_2_, showed a modest negative correlation over time; however, there was no significant difference in RCP/VAT between the younger and older groups. RCP/VAT is considered a more moderate indicator of the effect of aging than ΔVO_2_/weight RCP-VAT. The results suggest that the ability to excrete CO_2_ produced *in vivo* or to accumulate and buffer CO_2_ is less affected by aging.

### Respiratory Compensation Point/Ventilatory Anaerobic Threshold and ΔVO_2_ Respiratory Compensation Point-Ventilatory Anaerobic Threshold and Isocapnic Buffering Phase Time

For some IB phases that have been used in the past, the IB phase time can only be used for comparison in the same protocol. In addition, when the IB phase is expressed by ΔVO_2_/weight RCP-VAT, it is not possible to determine whether the decrease is only in that part of the whole exercise or the whole exercise, given that it is a cut-off of the oxygen uptake during exercise. In fact, as in a previous study (Carriere et al., 2019), peak VO_2_ and ΔVO_2_/weight RCP-VAT were correlated; however, since ΔVO_2_/weight RCP-VAT decreases with age, it is not suitable for comparison between groups of different ages. Conversely, the ratio of oxygen uptake, such as RCP/VAT, shows that the balance of exercise tolerance indices is maintained, though overall exercise tolerance decreased.

At low exercise intensities, oxidative phosphorylation is the main source of ATP production. As the exercise intensity increases, glycolysis increases, leading to lactate production and buffering. When the RCP is exceeded, lactic acidosis occurs. Since mitochondrial function including oxidative phosphorylation is not affected by aging or is only mildly affected (Lanza et al., 2005), VAT is considered to be less susceptible to the decline in mitochondrial function, except in certain diseases.

For a practical recommendation, by combining data with VAT and RCP VO_2_ and ΔVO_2_/weight RCP-VAT, we believe that RCP/VAT can possibly be used as a concise indicator for lactate buffering capacity and skeletal muscle composition ratio.

### Research Limitations

This study had several limitations. First, because the analysis was based on only exhaled gas analysis, hematological parameters such as blood levels of lactate and bicarbonate ions, as well as hemodynamics, could not be evaluated in parallel. RCP is associated with maximal lactate steady state, and exercise therapy improves skeletal muscle function (oxidative capacity); however, in this study, it was difficult to clarify what led to the increase and improvement in RCP/VAT with exercise therapy. Second, RCP/VAT is an index that can be influenced by either an increase or decrease in one of the indices; a decrease in VAT may result in an increase in RCP/VAT. Third, the relationship with prognosis, as shown by IB phase time and ΔVO_2_/weight RCP-VAT, is unclear. Fourth, the effect of the degree of training on the lactate buffering capacity is unknown because we did not investigate the activity level or the degree of training in all participants. Finally, due to the small number of cases in the 30–60 age range, there is insufficient analysis of trends by age and by age group.

## Conclusion

The ratio of oxygen uptake from VAT to RCP was not significantly lower in healthy older participants than in healthy younger participants. Although exercise tolerance decreases with age, age does not have a robust effect on lactate buffering capacity on exercise tolerance.

## Data Availability Statement

The raw data supporting the conclusions of this article will be made available by the authors, without undue reservation.

## Ethics Statement

Ethical review and approval was not required for the study on human participants in accordance with the local legislation and institutional requirements. Written informed consent for participation was not required for this study in accordance with the national legislation and the institutional requirements.

## Author Contributions

KK and MA: conceptualization and methodology. KK: data curation, formal analysis, project administration, software, validation, visualization, and writing – original draft. MA: funding acquisition, resources, and supervision. KK, KI, TK, and MM: investigation. KI, TK, MM, and MA: writing – review and editing. All authors contributed to the article and approved the submitted version.

## Conflict of Interest

The authors declare that the research was conducted in the absence of any commercial or financial relationships that could be construed as a potential conflict of interest.

## Publisher’s Note

All claims expressed in this article are solely those of the authors and do not necessarily represent those of their affiliated organizations, or those of the publisher, the editors and the reviewers. Any product that may be evaluated in this article, or claim that may be made by its manufacturer, is not guaranteed or endorsed by the publisher.
